# Supplementary material for: Statistical Mechanics of Electrowetting
Source: Entropy (Basel). 2024 Mar 22;26(4):276. doi: 10.3390/e26040276 (PMC11049600; doi:10.3390/e26040276)
Supplement: Supplementary file 1 [file entropy-26-00276-s001.zip › Table S1.pdf]

Nomenclature

α	dimensionless ratio $\alpha \equiv A_p/A_0$
$\bar{\sigma}$	mean field value of σ
$\bar{C}$	mean field capacitance
χ	probability of finding the drop above the central cell
$\chi^{\pm}$	χ in advance $(+)$ and recession $(-)$
χ_c	probability $\chi_c = \chi^{\pm}$ at the advancing $(+)$ or receding $(-)$ transitions
$\cos\theta^{\pm}$	contact angle cosine in advance $(+)$ and recession $(-)$
$\cos\theta_e$	Young contact angle cosine
$\cos\theta_{\varpi}$	advancing $(\varpi = +1)$ or receding $(\varpi = -1)$ contact angle cosine
ΔE	energy that a cell must receive to transition from one level to another
ΔE^*_{el}	dimensionless electrostatic energy
$\Delta E^*_{\mathrm{int}}$	dimensionless interfacial contributions to ΔE
ΔE_{el}	electrostatic energy that a cavity must spend to fill with conductive liquid
δ_c	combination of capacitances $\delta_c \equiv (C^*_{2_0} - C^*_{1_0})/C^*_{4_0}$ in Eq. (31)
ϵ	area fraction on the top surface $\epsilon \equiv A_0/(A_0 + A_s)$
Γ_n	interfacial energy between the central cell and its next-nearest edge cells
Γ_o	energy needed for the contact line to advance over the cavity, or deleted if it recedes
Γ_p	surface energy required to replace gas by liquid on cavity walls
$\gamma_{\ell g}$	liquid-gas interfacial energy, a.k.a. surface tension
γ_{gs}	gas-solid interfacial energy
$\gamma_{s\ell}$	solid-liquid interfacial energy
κ	drop interface curvature at the contact line
κ^*	dimensionless interface curvature $\kappa^* \equiv \kappa v_p/A_0$
λ	dimensionless ratio $\lambda \equiv A_n/A_0$
$\langle \delta G \rangle$	expected incremental change in drop Gibbs free energy
$\langle \delta G_c \rangle$	incremental change in drop Gibbs free energy due to electrostatics (Eq. 31)
$\langle \delta G_o \rangle$	incremental change in drop Gibbs free energy as the contact line passes over cavity openings (Eq. 30)
$\langle \delta G_s \rangle$	incremental change in drop Gibbs free energy as the contact line passes over flat solid parts of the texture (Eq. 29)
$\langle \delta G_{\mathcal{L}} \rangle$	incremental change in drop Gibbs free energy associated with latent energy (Eq. 32)
$\langle \sigma \rangle$	expected value of σ
$\langle C^* \rangle$	expected dimensionless capacitance
$\mathbb{H}$	Heaviside step function
$\mathcal{L}$	dimensionless latent energy change $\mathcal{L} = \Delta\langle E^* \rangle$ across the transition in expected value of unit cell energy
$\mathcal{L}^{\pm}$	dimensionless latent energy change in advance $(+)$ or recession $(-)$
ν	surface charge density
$\partial V/\partial y$	potential gradient perpendicular to the base
σ	Ising filling state variable
$\mathrm{d}A_{\ell g}$	change in the liquid-gas interfacial area $A_{\ell g}$ of the drop
$\mathrm{d}A_{gk}$	change in interfacial area A_{gk} between gas g around the drop and matter k (liquid, solid or gas) in unit cells

$dA_{k\ell}$	change in interfacial area $A_{k\ell}$ between drop liquid ℓ and matter k (liquid, solid or gas) in unit cells
$d\bar{G}$	change in Gibbs free energy of a drop over the textured surface
dG	change in drop Gibbs free energy
v_0	dielectric permittivity of vacuum
ξ	dimensionless square voltage in Eq. (1)
A	interfacial area
A_0	cavity opening area
A_n	combined cross-section areas linking the central cell to its adjacent neighbors
A_p	overall area of the solid walls and base of a cavity
A_s	pillar cross-section area
b	width of square pillar (Fig. 1)
b^*	dimensionless width $b^* = b/H$
C	capacitance
C^*	dimensionless capacitance $\equiv CH/K_s v_0(A_0 + A_s)$
$C^*_{i_j}$	dimensionless capacitance $C^*_i(n = j)$ of a unit cell in state i with j neighboring edge cavities of unlike filling
C^*_c	dimensionless lumped capacitance (Fig. 5)
C^*_d	dimensionless lumped capacitance (Figs. 2-5)
C^*_k	dimensionless lumped capacitance (Fig. 5)
C^*_m	dimensionless lumped capacitance (Fig. 4)
C^*_p	dimensionless lumped capacitance (Figs. 2, 3, 5)
C^*_q	dimensionless lumped capacitance (Figs. 2, 3, 5)
C^*_w	dimensionless lumped capacitance (Figs. 2-3)
C^*_ℓ	dimensionless lumped capacitance (Figs. 2-3)
$C^*_{i_0}$	dimensionless capacitance of a unit cell in state i with all neighboring edge cavities of like filling
d	gap between two adjacent pillars (Fig. 1)
d^*	dimensionless gap $d^* = d/H$
E	energy of a unit cell, a.k.a. Hamiltonian
E^*	dimensionless energy $E^* \equiv E/(A_0\gamma_{\ell g})$
E_c	electrostatic energy
H	overall solid sheet thickness, including pillars, if any (Fig. 1)
h	pillar height above the cavity bottom (Fig. 1)
K	material dielectric constant
k	subscript representing gas (g), liquid (ℓ), solid (s) or effective properties ($\bar{s}$) of the textured surface
K_s	material dielectric constant of the solid
n	number of edge cells around a cavity (Fig. 1)
p_i	five lumped-parameters $i = 1, 5$ predicting capacitances in Appendix A
$P_{1\,\text{or}\,2}(n)$	probability to have n edge cells in cases 1 or 2
U	voltage (DC) or root-mean-square voltage (AC) [13]
v_p	cavity volume
W	work of pressure

w	uniform thickness of the dielectric layer in the design of Krupenkin, et al [12]
Z_1^*	dimensionless lumped impedance (Fig. 2)
Z_2^*	dimensionless lumped impedance (Fig. 3)
Z_ℓ^*	dimensionless lumped impedance (Fig. 3)
Z_{ab}^*	dimensionless lumped impedance (Fig. 3)
Z_a^*	dimensionless lumped impedance (Fig. 3)
Z_{bc}^*	dimensionless lumped impedance (Fig. 3)
Z_b^*	dimensionless lumped impedance (Fig. 3)
Z_{ca}^*	dimensionless lumped impedance (Fig. 3)
Z_c^*	dimensionless lumped impedance (Fig. 3)
Z_f^*	dimensionless lumped impedance (Fig. 2)
Z_g^*	dimensionless lumped impedance (Fig. 2)
Z_r^*	dimensionless lumped impedance (Fig. 3)
Z_t^*	dimensionless lumped impedance (Fig. 2)
